# Development of prognostic indicator based on NAD+ metabolism related genes in glioma

**DOI:** 10.3389/fsurg.2023.1071259

**Published:** 2023-01-26

**Authors:** Xiao Chen, Wei Wu, Yichang Wang, Beichen Zhang, Haoyu Zhou, Jianyang Xiang, Xiaodong Li, Hai Yu, Xiaobin Bai, Wanfu Xie, Minxue Lian, Maode Wang, Jia Wang

**Affiliations:** ^1^ Department of Neurosurgery, The First Affiliated Hospital of Xi’an Jiaotong University, Xi’an, China; ^2^ Center for Brain Science, The First Affiliated Hospital of Xi’an Jiaotong University, Xi’an, China

**Keywords:** glioma, nicotinamide adenine dinucleotide, PARP9, BST1, NMNAT2, CD38, immune infiltration, bioinformatics

## Abstract

**Background:**

Studies have shown that Nicotinamide adenine dinucleotide (NAD+) metabolism can promote the occurrence and development of glioma. However, the specific effects and mechanisms of NAD+ metabolism in glioma are unclear and there were no systematic researches about NAD+ metabolism related genes to predict the survival of patients with glioma.

**Methods:**

The research was performed based on expression data of glioma cases in the Cancer Genome Atlas (TCGA) and Chinese Glioma Genome Atlas (CGGA) databases. Firstly, TCGA-glioma cases were classified into different subtypes based on 49 NAD+ metabolism-related genes (NMRGs) by consensus clustering. NAD+ metabolism-related differentially expressed genes (NMR-DEGs) were gotten by intersecting the 49 NMRGs and differentially expressed genes (DEGs) between normal and glioma samples. Then a risk model was built by Cox analysis and the least shrinkage and selection operator (LASSO) regression analysis. The validity of the model was verified by survival curves and receiver operating characteristic (ROC) curves. In addition, independent prognostic analysis of the risk model was performed by Cox analysis. Then, we also identified different immune cells, HLA family genes and immune checkpoints between high and low risk groups. Finally, the functions of model genes at single-cell level were also explored.

**Results:**

Consensus clustering classified glioma patients into two subtypes, and the overall survival (OS) of the two subtypes differed. A total of 11 NAD+ metabolism-related differentially expressed genes (NMR-DEGs) were screened by overlapping 5,995 differentially expressed genes (DEGs) and 49 NAD+ metabolism-related genes (NMRGs). Next, four model genes, PARP9, BST1, NMNAT2, and CD38, were obtained by Cox regression and least absolute shrinkage and selection operator (Lasso) regression analyses and to construct a risk model. The OS of high-risk group was lower. And the area under curves (AUCs) of Receiver operating characteristic (ROC) curves were >0.7 at 1, 3, and 5 years. Cox analysis showed that age, grade G3, grade G4, IDH status, ATRX status, BCR status, and risk Scores were reliable independent prognostic factors. In addition, three different immune cells, Mast cells activated, NK cells activated and B cells naive, 24 different HLA family genes, such as HLA-DPA1 and HLA-H, and 8 different immune checkpoints, such as ICOS, LAG3, and CD274, were found between the high and low risk groups. The model genes were significantly relevant with proliferation, cell differentiation, and apoptosis.

**Conclusion:**

The four genes, PARP9, BST1, NMNAT2, and CD38, might be important molecular biomarkers and therapeutic targets for glioma patients.

## Introduction

Glioma originates mainly from glial cells and is the most common primary brain tumor. It involves a broad category of central nervous system tumors, including astrocytoma, oligodendroglioma, and glioblastoma. Currently, the standard clinical strategy for glioma includes maximum surgical resection followed by radiotherapy, and temozolomide (TMZ) chemotherapy (1). However, the overall treatment effect was not ideal. Even with standardized treatment, the median survival time of glioblastoma multiforme (GBM) was only about 15 months, and the 5-year survival rate was less than 10% (2). Thus, considering the limited treatment strategies for glioma, there is an urgent need to develop reliable prognostic biomarkers and therapeutic targets.

Nicotinamide adenine dinucleotide (NAD+) is one of the most important co-enzymes (or co-factors) in oxidation-redox reactions, and NAD+ is also the core of energy metabolism (3, 4). Playing a key role in energy transduction and cell signal transduction, NAD+ can be transformed into NADP, NAADP and cADPR (4, 5). Moreover, NAD+ degradation products, such as nicotinamide and N-methyl nicotinamide, have also been considered as key regulators of energy metabolism, epigenetics and disease status (6–8). NAD+ pathway metabolites can also serve as substrates for a group of diverse enzymes (9, 10), including SARM1, ARTs, PARPs, CD38, sirtuins, and RNA polymerases, which were involved in several aspects of cellular homeostasis. Previous studies mainly focused on sugar metabolism, lipid metabolism, amino acid metabolism, nucleotide metabolism and energy metabolism. Interestingly, an increasing number of studies have revealed that NAD+ metabolism was closely related to the pathogenesis of many tumors (11, 12). For example, in GBM patients, the expression of NAMPT was associated with poor prognosis (13). More than that, NAD+ metabolism was reported to play an important role in tumor immunity (11, 14–16). But the mechanisms of NAD+ metabolism in glioma were unclear and there were no systematic researches about NAD+ metabolism related genes to predict the survival of patients with glioma. Different from previous researches, our study aimed to explore the prognostic value of NAD metabolism related genes in glioma by bioinformatics analysis.

Therefore, we used the data downloaded from the Cancer Genome Atlas (TCGA) in this study to perform bioinformatics analysis based on NAD+ metabolism related genes and then build a new risk model to predict the prognosis of glioma patients. Then we validate the model using Chinese Glioma Genome Atlas (CGGA). This model could find prognostic molecular markers and potential therapeutic targets for glioma patients, which provided an important reference value on selection of treatment strategies for clinicians, and also provided new ideas for the future basic research of glioma.

## Results

### Classification of glioma subtypes

**Figure 1:**
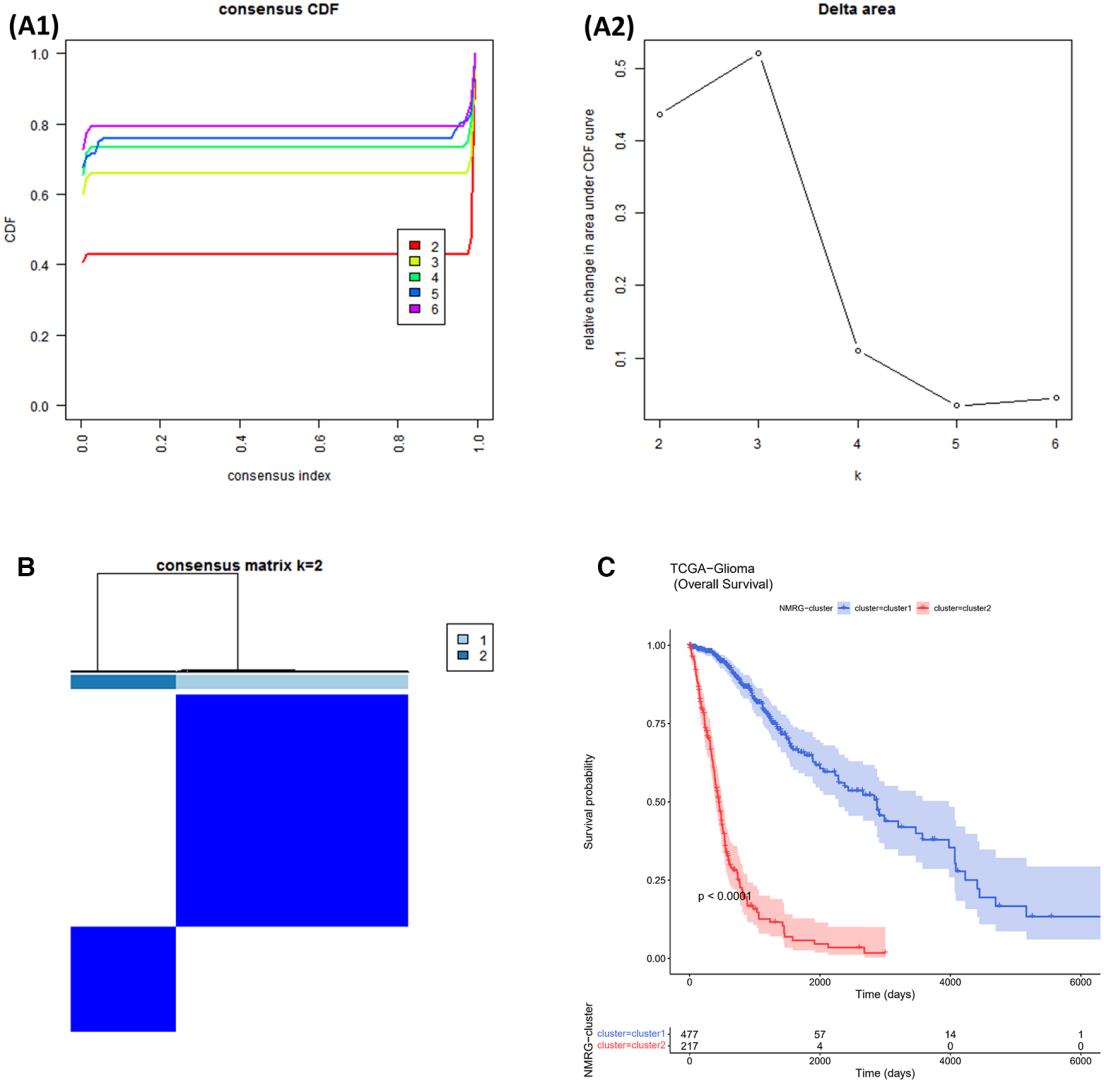
NMRGs classify glioma patients into different two subtypes. (**A1,A2**) Cumulative Distribution Function (CDF) plot for consistent clustering (**B**) consistency clustering heat map. The colours indicate the ease of clustering. (**C**) Survival curves for cluster1 and cluster2.

The consensus clustering results showed that the best clustering was achieved when *k* = 2. All glioma samples were classified into cluster 1 (*n* = 477) and cluster 2 (*n* = 217) (Figures 1A,B). In addition, the overall survival (OS) of cluster 1 patients was worse (*p* < 0.05). From the above, it is clear that NAD+ metabolism-related genes (NMRGs) can classify glioma patients into two subtypes with statistical difference in OS, indicating that NMRGs can affect the OS of glioma patients (Figure 1C).

### Identification of NAD+ metabolism-related differentially expressed genes (NMR-DEGs)

**Figure 2:**
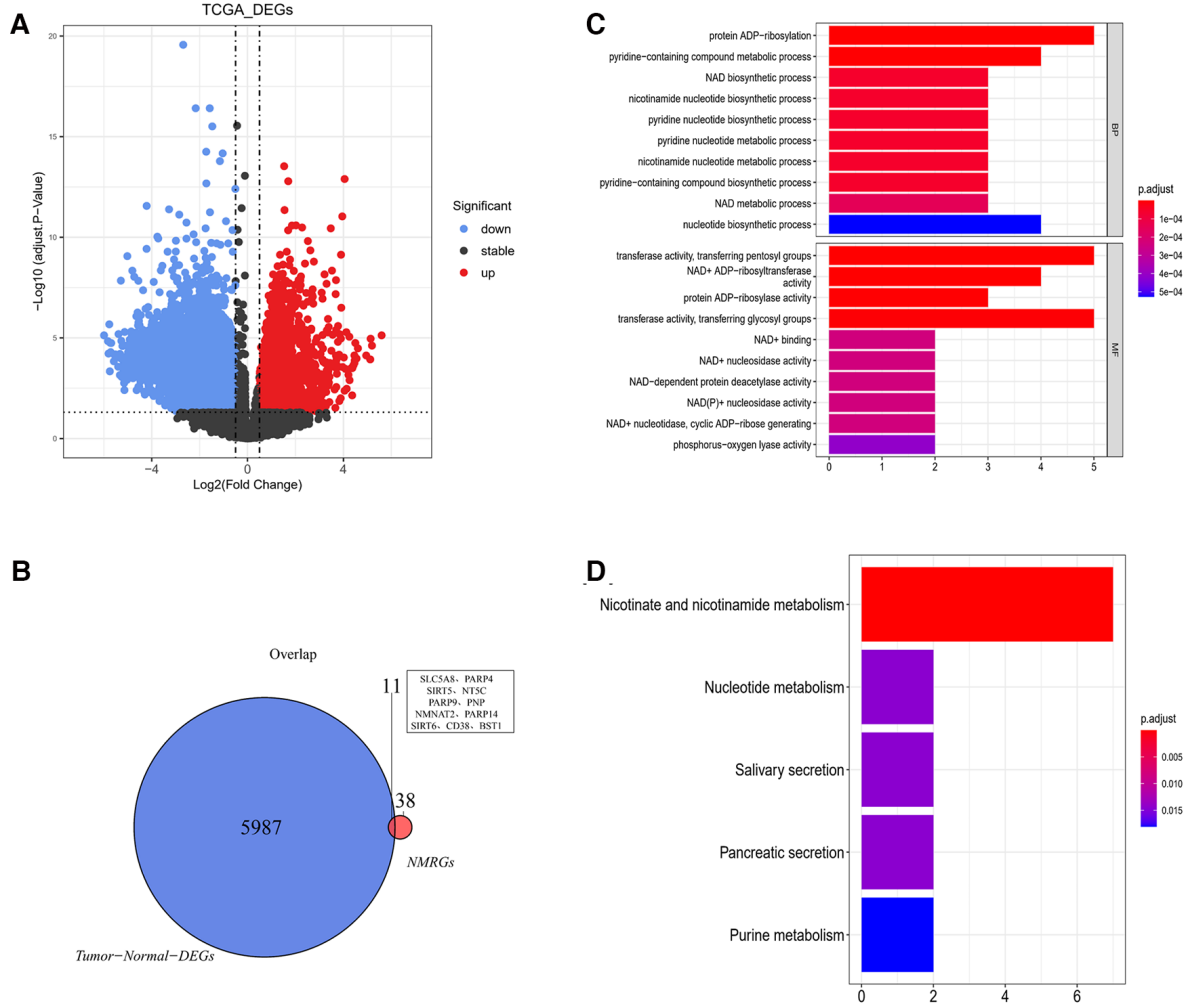
NAD+ metabolism-related differentially expressed genes were selected from glioma database. (**A**) Volcano map of differentially expressed gene analysis (DEGs) between glioma and normal samples. The horizontal coordinate log2FC indicates the difference multiplicity and the vertical coordinate indicates the confidence level - log10(adj. Pvalue). Each point in the graph represents a gene, with blue and red points representing significantly differentially expressed genes. Red dots indicate that their gene expression was up-regulated in the glioma samples and blue dots indicate that the gene was down-regulated in the glioma samples. (**B**) Venn diagram of differentially expressed genes with NMRGs. (**C,D**) Bar graphs of Gene ontology (GO) analysis and Kyoto Encyclopedia of Genes and Genomes (KEGG) analysis for NMR-DEGs.

5,998 DEGs were obtained between glioma and normal samples (Figure 2A and Supplementary Tables S1,S2). 11 NMR-DEGs were obtained after crossover analysis (Figure 2B and Table 1), and they were enriched in metabolic processes such as NAD+ metabolism, pyridine nucleotide metabolism, nucleotide metabolism, nicotinate and nicotinamide metabolism, as well as NAD+ biosynthesis process, pyridine nucleotide biosynthesis, and nucleotide biosynthesis. And NMR-DEGs were associated with NAD+ nucleotidase, circulating ADP-ribose-gluconeogenesis, NAD(P)+ nucleosidase activity, NAD+-dependent protein deacetylase activity, NAD+ binding transferase activity, NAD+ nucleosidase activity, NAD + ADP ribosyl transferase activity, transferase activity-transferring pentanediyl group and other pathways (Figures 2C,D).

**Table 1:** Differentially expressed genes between glioma and normal samples.

Symbol	logFC	AveExpr	*p*-Value	adj.P.Val	type
CD38	1.705	7.157	0.013	0.043	up
BST1	1.455	5.049	0.013	0.043	up
PARP9	1.401	9.663	0.002	0.010	up
PARP4	1.302	9.898	<0.001	<0.001	up
PARP14	1.216	9.867	0.003	0.015	up
PNP	1.060	9.265	0.002	0.010	up
NT5C	0.844	8.505	0.001	0.006	up
SIRT6	0.586	8.568	0.006	0.026	up
SIRT5	−0.690	8.305	0.0002	0.001	down
SLC5A8	−1.077	0.221	<0.001	<0.001	down
NMNAT2	−2.381	9.746	0.003	0.013	down

### Building and validating of risk model

**Figure 3:**
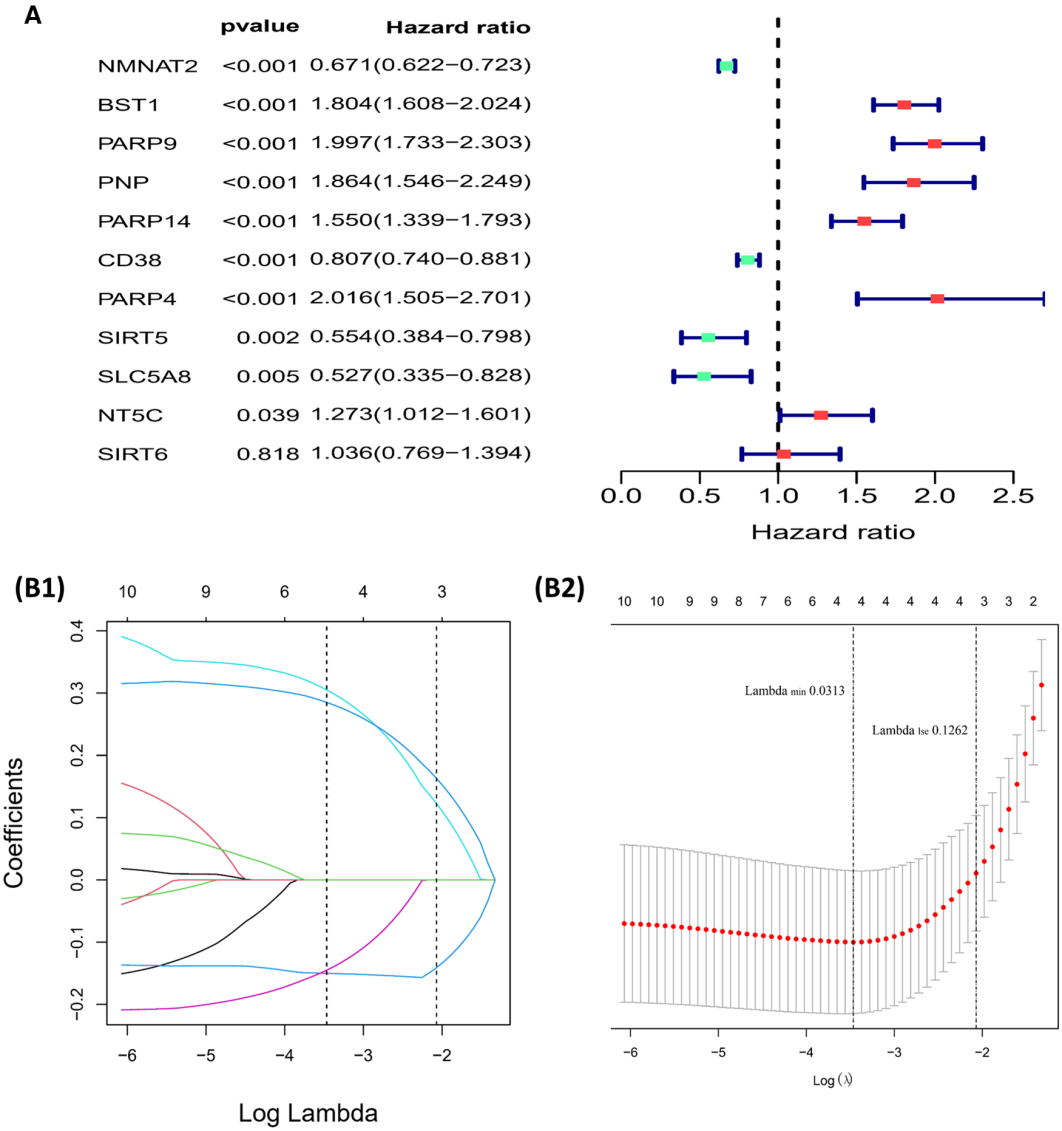
Building of the risk model (**A**) forest plot for univariate cox regression analysis. (**B1,B2**) Plot of gene coefficients for Lasso regressions. Error plots for 10-fold cross-validation.

**Figure 4:**
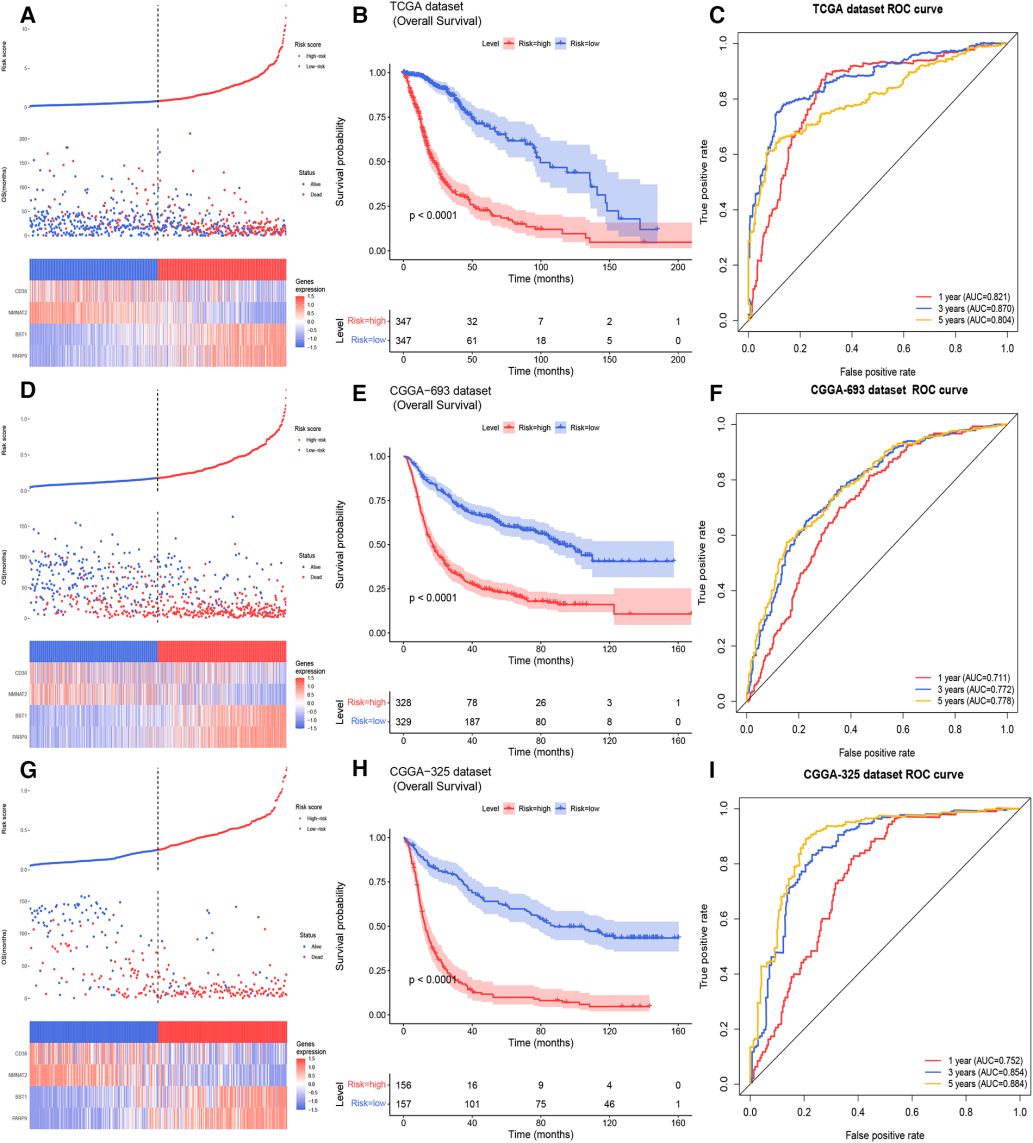
Building and validating of a risk model (**A**) the risk curve, scatter diagram, and model gene expression heat map of the training cohort (**B**) survival curves of the high- and low- risk groups of the training cohort. (**C**) The ROC curves of glioma patients in training cohort for 1, 3, 5 years (**D**) the risk curve, scatter diagram and model gene expression heat map of the CGGA 693 cohort. (**E**) Survival curves demonstrating that glioma cases in the high- and low- risk groups of the CGGA 693 cohort. (**F**) The ROC curves of glioma patients in the CGGA 693 cohort. (**G**) The risk curve, scatter diagram, and model gene expression heat map of the CGGA 325 cohort. (**H**) Survival curves for glioma cases of the CGGA 325 cohort in the two risk groups. (**I**) The ROC curves of glioma patients in CGGA 325 cohort.

11 NMRGs were obtained (Figure 3A). When lambda min = 0.0313, PARP9, BST1, NMNAT2, and CD38 were obtained (Figure 3B and Table 2). Then the glioma cases were classified into a high-risk group (*n* = 347) and a low-risk group (*n* = 347) (Median Risk score = 0.8125), and cases in the high-risk group had a lower OS (Figures 4A,B). The area under curves (AUCs) of the ROC curves in the training cohort were all greater than 0.8 (Figure 4C). In the validation set, the OS was worse in the high-risk group, and the AUCs of the 1, 3, 5 years in the ROC curves were >0.7, consistent with the results of TCGA (Figures 4D–F).

**Table 2:** Coef of 4 model genes in lasso analysis.

gene symbol	coef	exp (coef)	se (coef)	*z*	*p*
NMNAT2	−0.15	0.86	0.05	−3	0.006
PARP9	0.37	1.45	0.1	4	0.0001
CD38	−0.21	0.81	0.05	−4	0.0001
BST1	0.33	1.39	0.08	4	0.00002

### Correlation analysis of risk model and clinical factors

**Figure 5:**
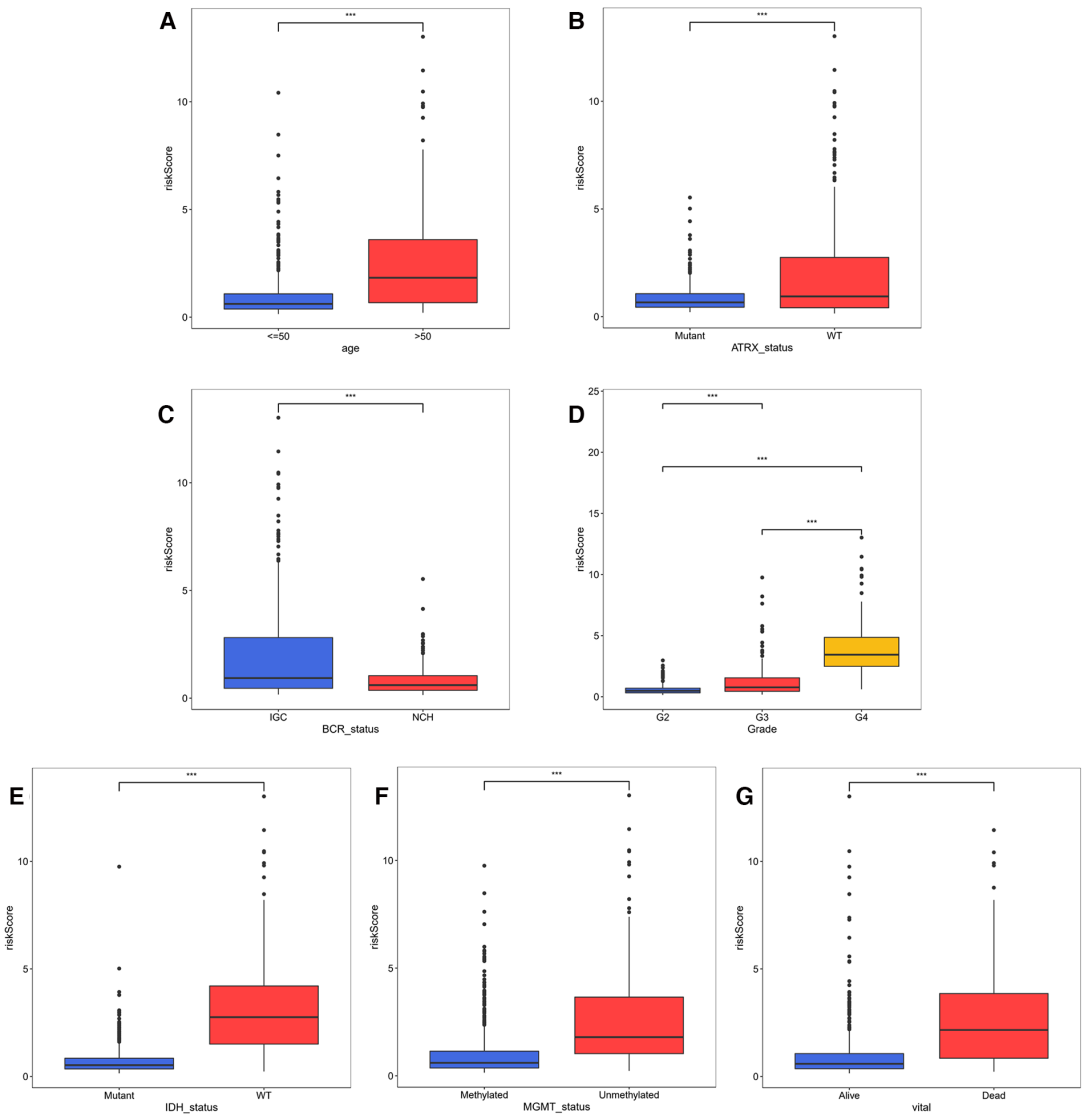
Correlation analysis of risk model and clinical factors (**A–D**) comparison of risk scores of different clinical subgroups of TCGA glioma patients.

**Figure 6:**
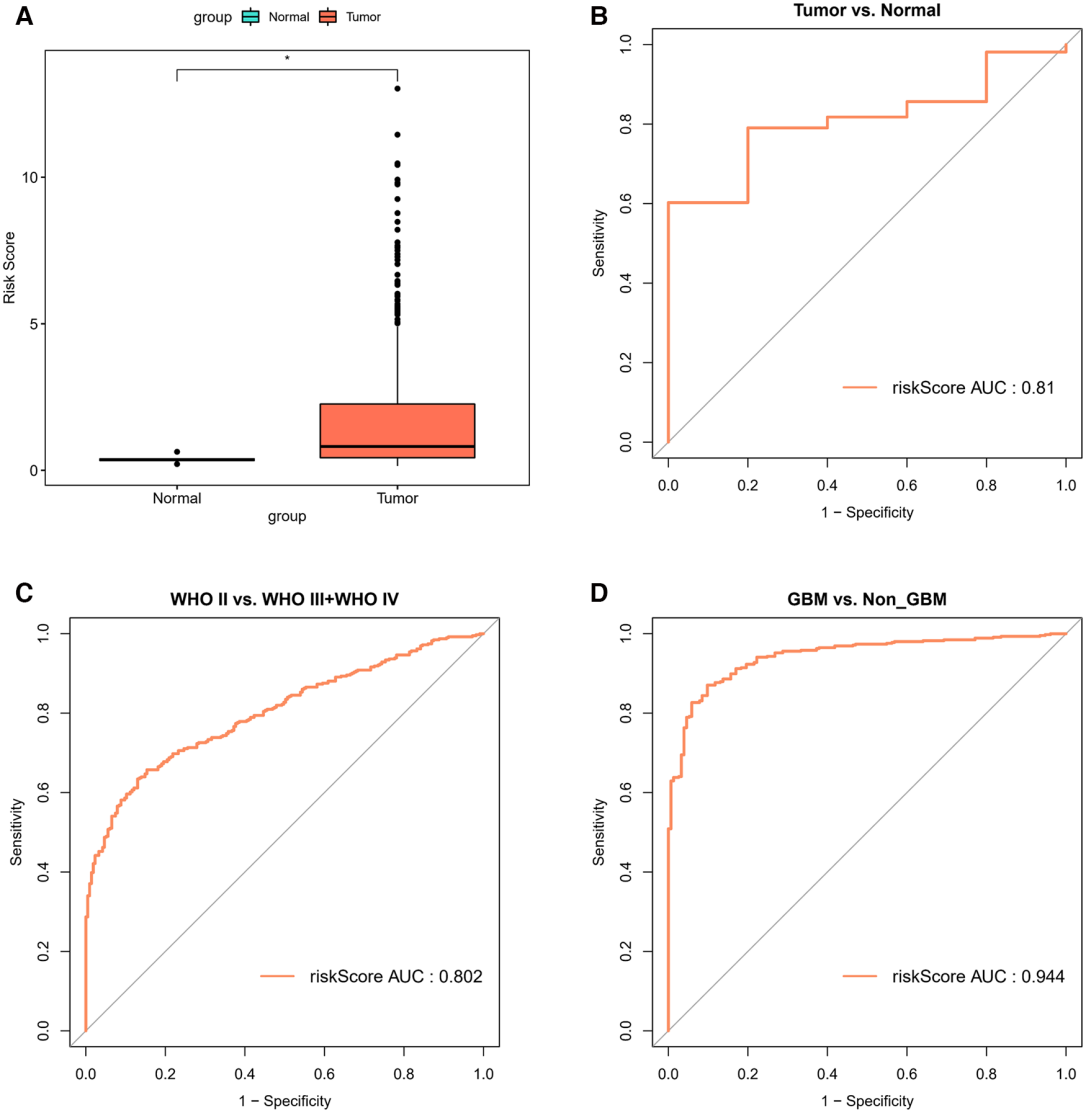
Correlation analysis of risk model and clinical factors (**A**) comparison of differences in risk scores between tumor and normal samples. (**B**) The ROC curves for risk score differentiation between Tumor and Normal samples. (**C**) The ROC curves for Grade4 and Grade2 + Grade3 samples with risk score differentiation. (**D**) The ROC curves for GBM and non-GBM samples with risk score differentiation.

In the TCGA-glioma dataset, risk Scores were significantly correlated with age, Grade, survival status, IDH status, MGMT status, ATRX status and BCR status. And risk Scores for age, Grade, BCR status, IDH status, MGMT status, ATRX status, and BCR status were significantly different (Table 3). In the CGGA-glioma dataset, risk Scores for gender were significantly different (Table 4). In addition, the risk Scores for age (>50 vs. ≤50), Grade (G2/G3/G4), BCR status (NCH vs. IGC), IDH status (WT vs. Mutant), MGMT status (Unmethylated vs. Methylated) and ATRX status (WT vs. Mutant) were significantly different (Figure 5). Moreover, the risk Score was higher in tumor tissue (Figure 6A). The ROC curves demonstrated that the risk Score could significantly distinguish between tumor and normal samples (AUC = 0.81, Figure 6B), and it had a strong ability to distinguish between high-grade glioma (G3 + G4) and low-grade glioma (G2) (AUC = 0.802, Figure 6C). Besides, the risk Score could also distinguish between GBM and non-GBM (AUC = 0.922, Figure 6D).

**Table 3:** Risk and clinical data in TCGA.

Variables	Risk in TCGA			*p*-value
	Total	high	low	
**age (year)**				
Mean (SD)	47.3 (±15.3)	52.6 (±15.4)	41.9 (±13.2)	<0.001
**Gender**				
Female	254 (41.7%)	125 (40.3%)	129 (43.1%)	0.51
Male	355 (58.3%)	185 (59.7%)	170 (56.9%)	
**Vital**				
Alive	428 (61.7%)	146 (42.1%)	282 (81.3%)	<0.001
Dead	266 (38.3%)	201 (57.9%)	65 (18.7%)	
**Neoplasm Histologic Grade**				
G2	215 (35.3%)	43 (13.9%)	172 (57.5%)	<0.001
G3	241 (39.6%)	119 (38.4%)	122 (40.8%)	
G4	153 (25.1%)	148 (47.7%)	5 (1.7%)	
**IDH status**				
Mutant	427 (64.7%)	112 (34.7%)	315 (93.5%)	<0.001
WT	233 (35.3%)	211 (65.3%)	22 (6.5%)	
**MGMT promoter status**				
Methylated	476 (74.8%)	170 (56.9%)	306 (90.8%)	<0.001
Unmethylated	160 (25.2%)	129 (43.1%)	31 (9.2%)	
**ATRX status**				
Mutant	195 (29.7%)	71 (22.2%)	124 (36.8%)	<0.001
WT	462 (70.3%)	249 (77.8%)	213 (63.2%)	
**DAXX status**				
Mutant	2 (0.3%)	2 (0.6%)	0 (0.0%)	0.24
WT	654 (99.7%)	318 (99.4%)	336 (100.0%)	
**BCR Status**				
IGC	504 (75.6%)	271 (82.9%)	233 (68.5%)	<0.001
NCH	163 (24.4%)	56 (17.1%)	107 (31.5%)	

**Table 4:** Risk and clinical data in CGGA.

Variable	Risk			*p*-value
	Total	high	low	
**age (year)**				
Mean (SD)	43.5 (±12.4)	44.1 (±12.6)	42.8 (±12.2)	0.3
**gender**				
Female	283 (43.1%)	139 (42.4%)	144 (42.2%)	0.02
Male	374 (56.9%)	189 (57.6%)	185 (54.7%)	
**Vital**				
Alive	263 (40.0%)	128 (39.0%)	135 (41.0%)	0.63
Dead	394 (60.0%)	200 (61.0%)	194 (59.0%)	
**Neoplasm Histologic Grade**				
WHO II	172 (26.2%)	85 (25.9%)	87 (26.4%)	0.98
WHO III	248 (37.7%)	125 (38.1%)	123 (37.4%)	
WHO IV	237 (36.1%)	118 (36.0%)	119 (36.2%)	
**IDH status**				
Mutant	333 (54.7%)	153 (52.4%)	180 (56.8%)	0.29
Wildtype	276 (45.3%)	139 (47.6%)	137 (43.2%)	
**MGMT promoter status**				
methylated	304 (58.2%)	190 (59.2%)	114 (56.7%)	0.59
un-methylated	218 (41.8%)	131 (40.8%)	87 (43.3%)	
**1p19q_codeletion_status**				
Codel	137 (23.2%)	68 (23.1%)	69 (23.2%)	1
Non-codel	454 (76.8%)	226 (76.9%)	228 (76.8%)	

### Independent prognostic analysis of risk model

**Figure 7:**
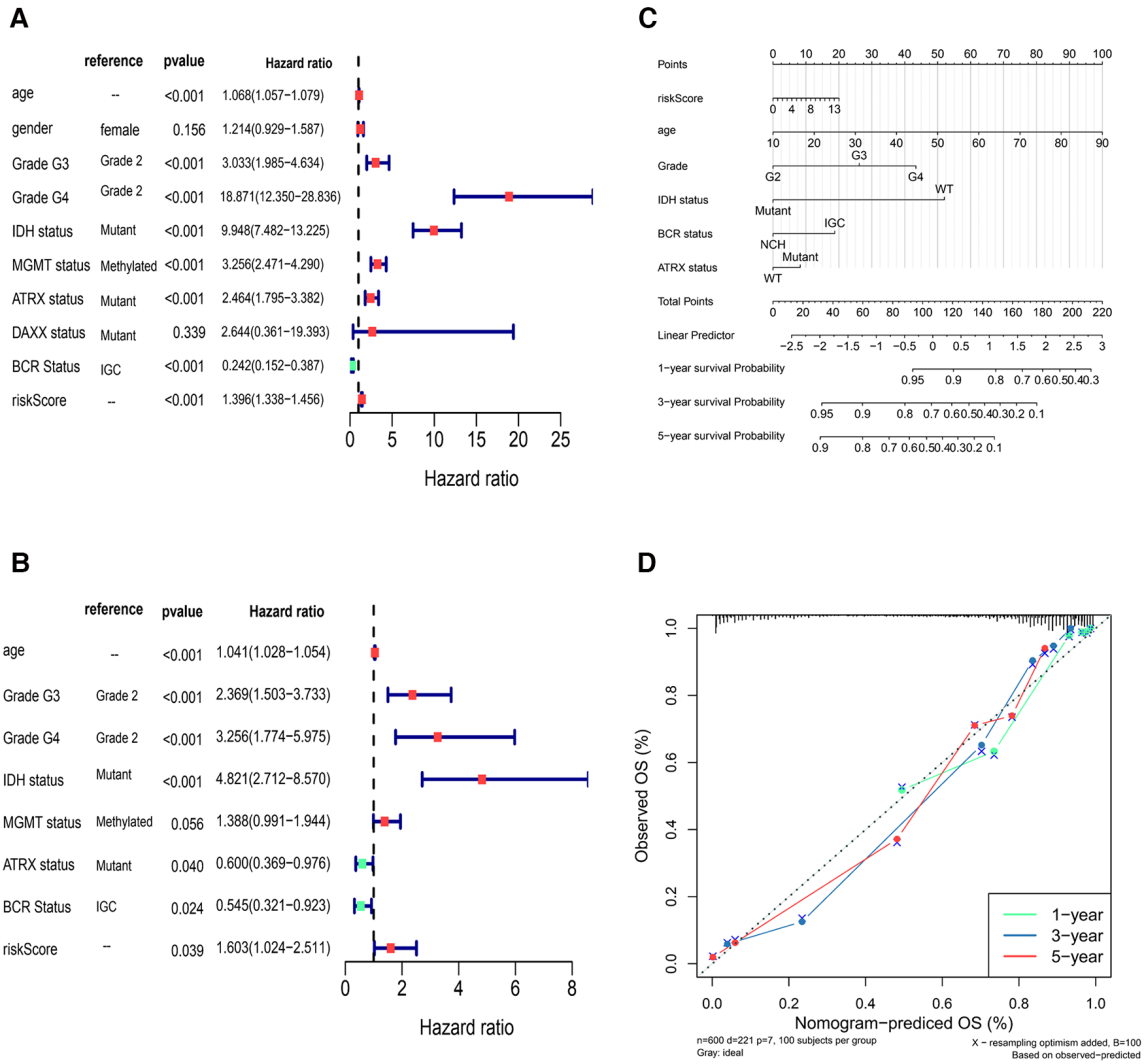
Independent prognostic analysis of risk model (**A,B**) univariate Cox and multivariate Cox independent prognostic analysis (**C**) the nomogram in TCGA glioma sample to predict 1, 3 and 5-year survival rate (the C-index was 0.8617) (**D**) the correction curve of nomogram.

The results showed that age, Grade, IDH status, ATRX status, BCR Status, and risk Score in Cox analysis were all *p* < 0.05 (Figures 7A,B). The nomogram in TCGA glioma samples was shown in Figure 7C, and the C-index was 0.8617, which indicated that the model had a better prediction effect. The correction curve of nomogram was shown in Figure 7D, and the closer the slope was to 1, the more accurate the prediction was. The accuracy of this risk model in predicting the OS of cases was high, indicating that the constructed prediction model could be a valid model.

### Correlation of risk score with KEGG pathway

**Figure 8:**
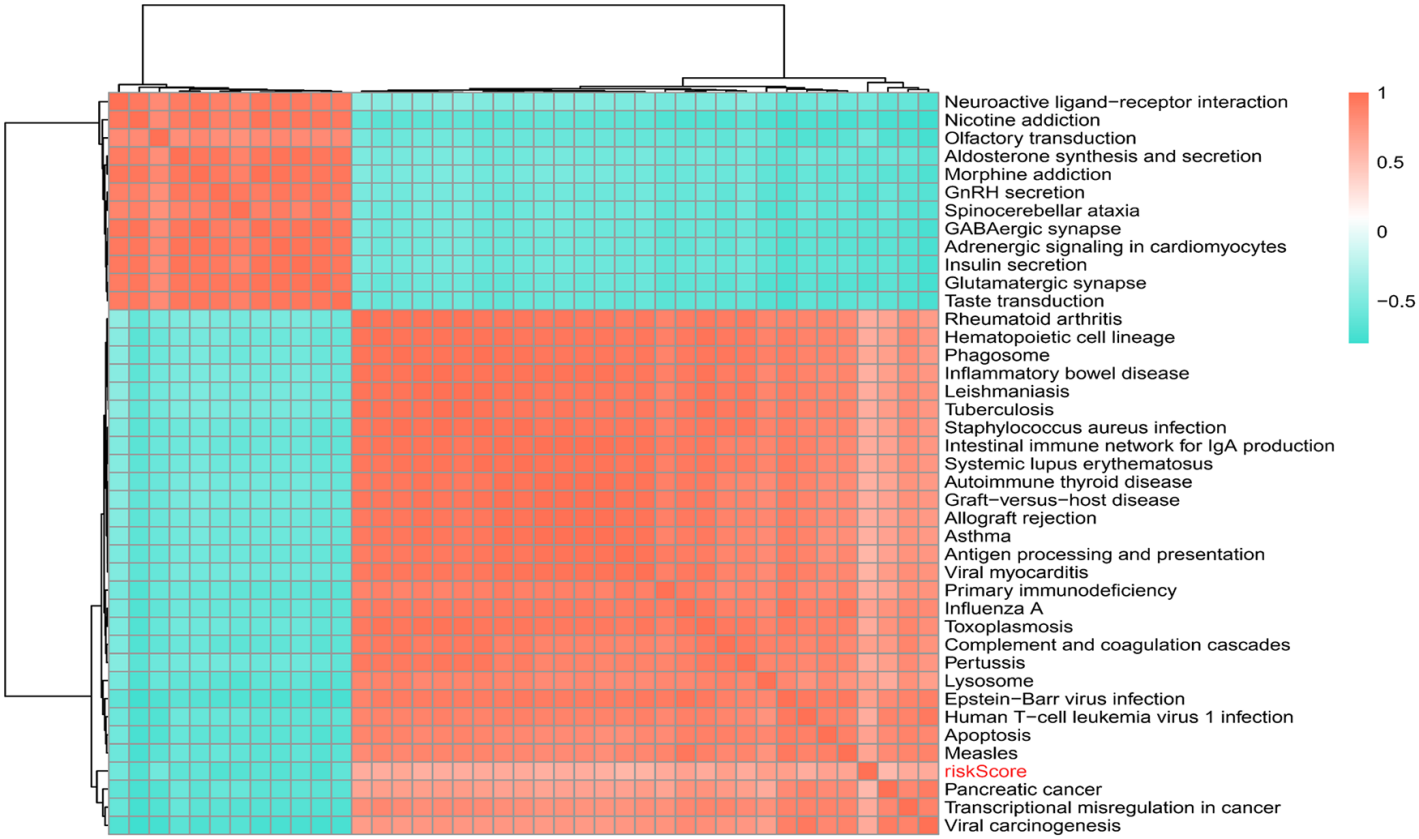
Correlation of risk scores with KEGG pathway.

309 pathways were significantly different, 40 pathways were connected with risk Score, 12 of which were positively connected with risk Score and 28 were negatively connected with risk score (Supplementary Table S3). The heat map of risk score and KEGG pathway was shown in Figure 8. As could be seen from the figure, all the pathways were positively connected with risk Score except for the Lysosome pathway, which was negatively connected with risk Score. Moreover, neuroactive ligand-receptor interactions, aldosterone synthesis and secretion, GnRH secretion, GABAergic synapses, adrenergic signaling pathway in cardiac myocytes, insulin secretion, and glutamatergic synapses were significantly correlated with risk Score.

### Impact of risk model on immune heterogeneity

**Figure 9:**
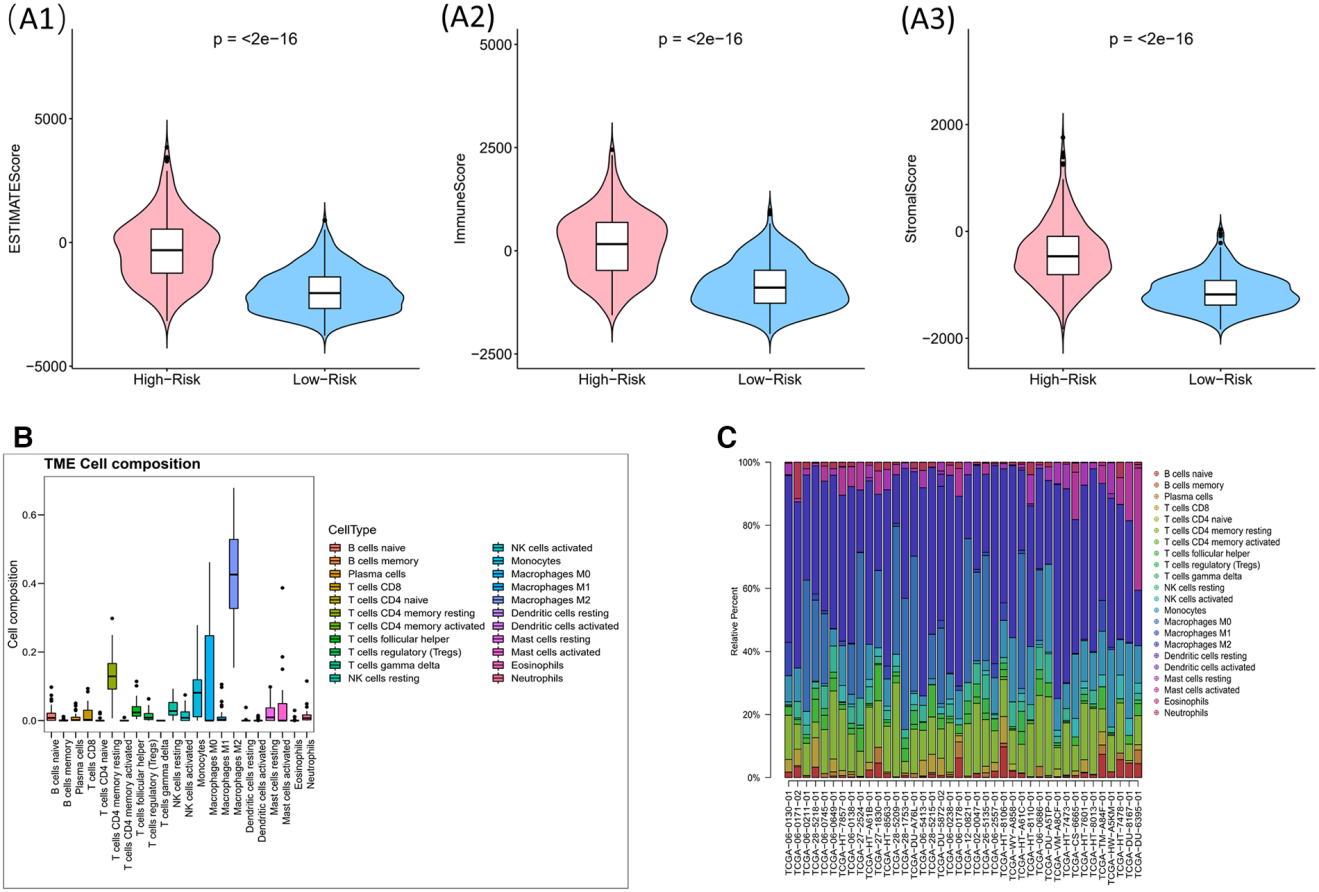
Impact of risk model on immune heterogeneity (**A**) violin diagram of immune scores, stromal scores and ESTIMATE scores for high and low risk groups. The horizontal coordinates represent the groupings (high-/low- risk groups) and the vertical coordinates represent the different scores, blue for the low risk group and pink for the high risk group. (**B,C**) The box line plots and stacked bar graphs show the abundance of immune cells.

**Figure 10:**
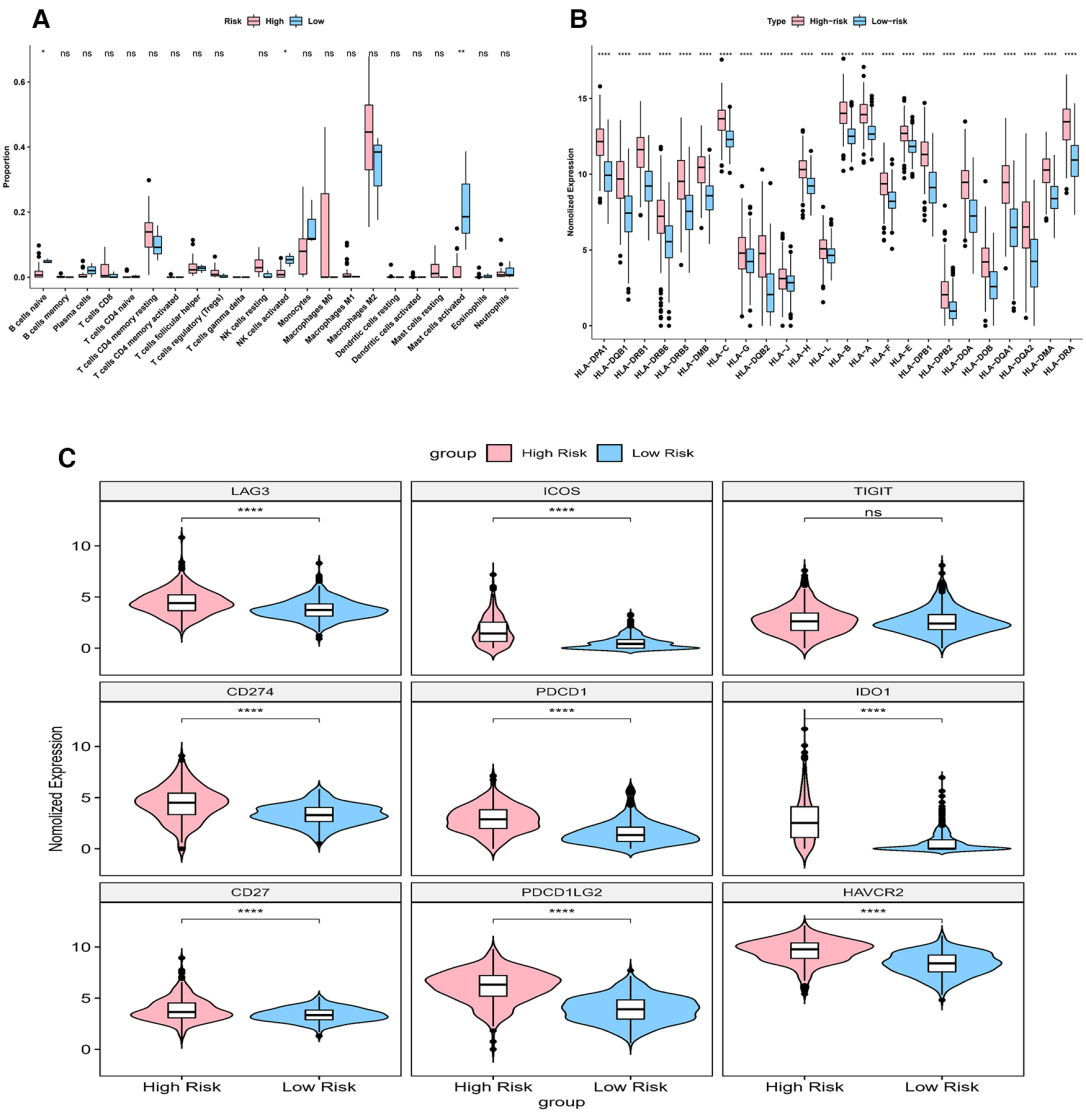
Impact of risk model on immune heterogeneity (**A**) the box line plots comparing the difference of immune cells between high and low risk groups. (**B**) The box line plots comparing the difference of HLA between high and low risk groups.

Immune score, stromal score and ESTIMATE score were higher in high-risk group (*p* < 0.05) (Figure 9A). The CIBERSORT algorithm calculated the proportion of each immune cell and the corresponding statistical values. After excluding samples with *p* > 0.05, 37 samples remained in the high-risk group and 3 samples remained in the low-risk group. The box line plots and stacked bar graphs plotted for the abundance of immune cells were shown in Figures 9B,C. Mast cells activated, NK cells activated, and B cells naive were less in the high-risk group (Figure 10A). All 24 HLA family genes were higher in the high-risk group (Figure 10B). Except for TIGIT, all the immune checkpoints were higher in the high-risk group (Figure 10C).

### Single-cell functional analysis of model genes

**Figure 11:**
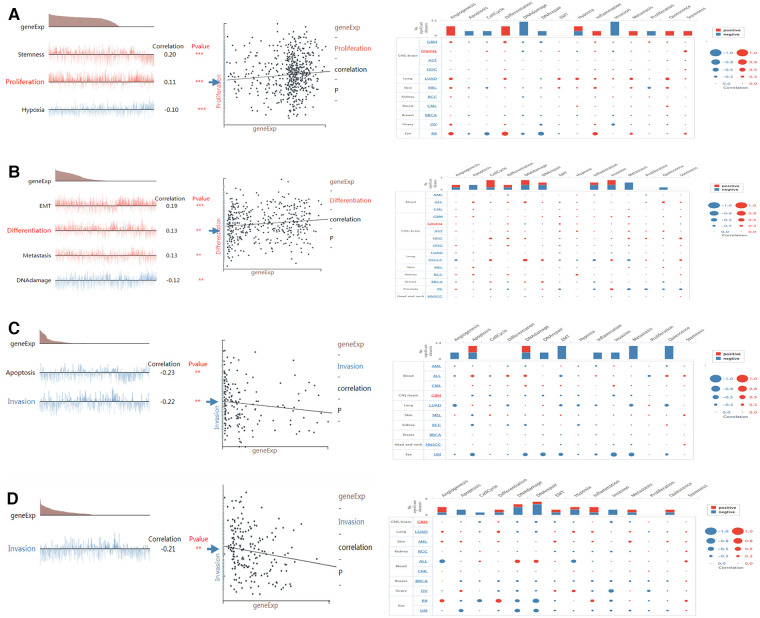
Single-cell functional analysis of model genes (**A–D**) functional status of (**A**) NMNAT2, (**B**) PARP9, (**C**) CD38, (**D**) BST1.

CancerSEA can provide insight into the model genes in individual glioma cell. NMNAT2 was positively connected with stemness and proliferation, and negatively connected with hypoxia (Figure 11A). PARP9 was significantly positively connected with EMT, cell metastasis, cell differentiation and negatively connected with DNA damage (Figure 11B). CD38 was negatively connected with both cell invasion and apoptosis (Figure 11C). BST1 was negatively connected with cell invasion (Figure 11D).

## Discussion

As the most common neuroepithelial tumor of the central nervous system, gliomas are a heterogeneous group of central nervous system tumors (17). A unique feature of glioma cells is the high demand for energetic molecules namely adenosine triphosphate (ATP), to sustain their rapid growth rate and the biosynthesis of DNA and proteins (18, 19). Products of NAD+ degradation, NAD+ pathway metabolites and many molecules converted from NAD+ were closely associated with the development and progression of gliomas (12, 14, 20–22). Nevertheless, there were no systematic researches about NAD+ metabolism related genes to predict the survival of patients with glioma. Therefore, it was necessary to establish a new glioma prognostic model based on NAD+ metabolism related genes to verify the prognostic molecular markers and potential therapeutic targets for glioma patients.

Currently, numerous glioma prognostic models have been reported. These glioma prognosis models can be divided into the following types (23): RNA models, methylation models, other multimolecular models. However, due to limitations, such as the lack of external validation, lack of gold markers, not providing quantitative results or absolute risk stratification, difficulties of data collection, the complexity of analysis, and low adherence to complete and transparent reporting, these models are still not recommended for clinical application (23, 24). The NAD+-related model constructed in this study contained only four model genes, it passed internal validation and external validation, was an accurate glioma model.

In this study, based on consensus cluster analysis of 42 NAD related genes (NMRGs), we first divided glioma patients into cluster 1 and cluster 2, with significant survival difference between the two clusters. Then, based on the differential expression analysis of genes between the two groups, Cox analysis and LASSO regression analysis, we further screened out four genes significantly related to the survival of glioma patients, and then established a risk scoring model and nomogram to evaluate their prognostic prediction and clinical application value. Through the correlation analysis between risk score and immune invasion, immunotherapy, KEGG pathway and single cell function enrichment analysis, we further explored the potential functions and signal pathways of the four model genes involved in glioma, with a view to providing more candidate targets for the treatment of glioma patients.

As one of the PARP family, PARP9 was highly expressed in gliomas and high PARP9 expression was associated with poor prognosis and clinicopathological features (15), which supported our findings. In addition, it was shown that PARP9 played an important role in the immune microenvironment of glioma and that PARP9 may be a prepotential immunotherapeutic target for glioma (15). CD38 mediated intracellular ATP levels and glioma cell survival (25). Moreover, CD38 deficiency regulated microglia activation through microglia-associated mechanisms to attenuate glioma progression, and it could modulate tumor-associated microglia/macrophage characteristics (16), which was also consistent with our study. However, to the best of our knowledge, BST1 and NMNAT2 were found to be closely associated with glioma for the first time. BST1 regulated nicotinamide riboside metabolism through glycohydrolase and base exchange activity, which has beneficial effects on aging and aging-related diseases (26). NMNAT2 was a target for the treatment of many malignancies. For example, upregulation of NMNAT2 was associated with the presence, depth and stage of colorectal cancer (27). Furthermore, specific deletion of NMNAT2 in mouse oocytes interfered with meiotic apparatus assembly and metabolic activity, where supplementation with niacin or forced expression of NMNAT2 in aging oocytes during *in vitro* culture reduced reactive oxygen species (ROS) production and the incidence of spindle/chromosome defects (28). Therefore, it is speculated that NMNAT2 may also act on glioma through NAD+ metabolism.

Some scholars reported that glioma patients with high immune scores and stromal scores were correlated with increased malignancy and reduced survival (29, 30). Our result, which showed that immune scores, stromal scores and ESTIMATE scores were higher in high-risk group than in low-risk group, was consistent with these reports. The majority of the non-neoplastic cells in gliomas are glioma-associated microglia/macrophages (GAMs), comprised macrophages of peripheral origin and brain-intrinsic microglia, which supported tumor progression (31). GAMs are usually divided into two categories, macrophages M1 and macrophages M2. Macrophages M2 are the most abundant immune cells within the glioma stroma (32, 33). Our study showed that Mast cells activated, NK cells activated, and B cells naive were less in the high-risk group, which supported this view. In addition, Tanzhu et al. obtained a similar result (34).

HLA family genes are essential for immunosurveillance and cancer immunotherapy (35, 36). Many HLA molecules, for example, HLA-E, HLA-F, HLA-G, HLA-H and HLA-DR were reported to be overexpressed on cancer cells (35–38). In our model, all 24 HLA family genes were higher in the high-risk group. More data is needed to verify it in the future. In general, most immune checkpoints (including PD-1, TIM-3, CD96, PDCD1, IDO1, PDCD1LG2, and CTLA-4) were highly expressed in glioma cells (39–42). Higher expressions of immune checkpoints were observed in more severe grades of glioma, and this indicated that it was linked to a worse prognosis (39–42). Our result showed that all the immune checkpoints were high in the high-risk group except for TIGIT, suggesting that they synergistically regulated the immune response in the tumor microenvironment. These findings opened up new possibilities for the treatment of gliomas.

An enquiry into the functional status of biomarkers in glioma showed that PARP9 was positively associated with EMT, cell metastasis and cell differentiation and negatively associated with DNA damage. It has been shown that PARP9 was essential for B cell development (43). In addition, the PARP inhibitor veliparib could act on DNA damage repair in prostate cancer cells (44), which supported our study. CD38 showed a significant negative correlation with both cell invasion and apoptosis. Previous studies suggested that CD38 was involved in cell differentiation and inflammatory processes and played a key role in the inflammatory process of autoimmunity (45, 46). NMNAT2 was significantly and positively associated with stemness and proliferation. Wu et al. found that NAD+ deficiency due to reduced NMNAT2 expression affected cell metabolism and meiosis (28), which provided evidence for our study.

There are several limitations in our study. First, the amount of data used in the analysis is not large, so our results may have certain deviation. More data is needed to validate this model in the future. Second, it is the result of bioinformatics analysis without experimental verification. More basic experiments are required to verify the specific mechanism of these genes in glioma. Third, more prospective studies are needed to prove the prognostic function of the four genes. In the future, we will also collect more clinical samples for in-depth *in vitro* and *in vivo* experimental research to further verify the relevant mechanisms of the four genes in glioma.

## Conclusion

For the first time we built a NAD+ metabolism related risk model, The model could predict the prognosis of glioma patients from another new perspective. Most importantly, it proved that CD38, NMNAT2, PARP9, and BST1 might be important prognostic molecular markers and potential therapeutic targets for glioma patients.

## Methods

### Data source

We acquired the expression data and clinical data of glioma from the TCGA and CGGA databases (Table 5). The TCGA database contains 697 glioma and 5 normal samples, of which 694 glioma samples had survival information. The CGGA 693 and CGGA 325 datasets contain 657 and 313 glioma samples with survival information, respectively. In addition, NMRGs were acquired from the Kyoto Encyclopedia of Genes and Genomes and the Reactome databases (Table 5). Finally, 49 NMRGs were obtained by combined genes obtained from the two databases (47).

**Table 5:** Data source.

Database	Website	Name
TCGA	https://portal.gdc.cancer.gov/	
CGGA	http://www.cgga.org.cn/	
KEGG	https://www.kegg.jp/	Pathway: hsa00760
Reactome	https://reactome.org/	R-HSA-196807

### Classification of glioma subtypes

Consensus clustering is a common method for classifying cancer subtypes (48). This study used “ConsensusClusterPlus” package (version 1.54.0) (49) to execute consensus clustering based on 42 NMRGs (Seven genes were not expressed in the Glioma samples) in the TCGA-glioma dataset. Then the survival analysis of different subtypes was performed by “survival” package (version 3.2–11).

### Screening of NMR-DEGs

The differentially expressed genes (DEGs) between glioma and normal samples were acquired by “limma” package in TCGA-glioma database (version 3.46.0) (50). Next, the DEGs were intersected with the NMRGs using the “VennDiagram” package (version 1.6.20) (51) to obtain NMR-DEGs. Then, functional enrichment analysis of NMR-DEGs was performed based on gene ontology (GO) and KEGG databases using the “clusterProfiler” package (version 3.18.0) (52). The enrichment results were also visualized by plotting bar graphs using the “enrichplot” package (version 1.10.2).

### Building and validating of a risk model

In this study, the 694 glioma cases from the TCGA database were used as the training cohort and the 657 cases from the CGGA database were used as the validation cohort to construct and validate the risk model. First, we extracted the expression data of NMR-DEGs in the training cohort for univariate Cox analysis. Then the factors with *p* < 0.05 were subjected to the least shrinkage and selection operator (Lasso) regression analysis. The risk Scores of glioma cases were counted using the following formula.


$$\mathrm{risk}\;\mathrm{score}=\overset{n}{\underset{n=1}{\sum }}\left(\mathrm{coe}{\;f}_{i}\times x_{i}\right)$$

The median value of risk Score was used as the boundary to classify the glioma cases into high and low risk groups. Then risk curves were plotted, and survival curves and receiver operating characteristic (ROC) curves were drawn by “survminer” (version 0.4.8) and “survivalROC” package (version 1.0.3) respectively. Also, we plotted risk curves, survival curves, and ROC curves in the validation set.

### Correlation analysis of risk model and clinical factors

To further investigate the relationship between clinicopathological characteristics and risk model, age, gender, survival status, Grade, IDH status, MGMT status, ATRX status, DAXX status, and BCR status were correlated with risk Scores in the training and validation cohorts. First, the number of patients with different clinical subtypes was compared between the high and low risk groups, and then the risk Scores were compared in the training set with different clinical information using rank sum tests. In addition, according to the abundance of model genes and their risk coefficient, the risk Score of normal samples was calculated, and the risk Score between tumor and normal samples was compared by the rank-sum test. Finally, the ROC curves were used to investigate the ability of the risk score to distinguish tumor tissue from normal tissue, and to distinguish between different Grades.

### Independent prognostic analysis of risk model

To discovery the independent prognosis of clinicopathological characteristics, risk scores and clinicopathological factors such as age, gender, Grade, IDH status, MGMT status, ATRX status, DAXX status, and BCR status were included in the risk model for Cox analyses in the training set. Next, a nomogram plot predicting survival of glioma patients was constructed. Finally, a calibration curve was plotted.

### Correlation of risk scores with KEGG pathway

We performed GSVA enrichment analysis based on KEGG pathway for all genes using “GSVA” package to find the functional difference between high and low risk groups (53). First, we obtained the enrichment scores in each pathway for the samples, and then performed a differential analysis of the pathways by the “limma” package with the screening condition of adjust *p *< 0.05. Next, we executed a correlation analysis of the differential pathways with the risk scores by setting the screening condition of |cor| > 0.6 and *p* < 0.01.

### Impact of risk model on immune heterogeneity

The immune status difference between high and low risk groups were executed by ESTIMATE analysis. The Immune Score, Stromal Score, and the combined ESTIMATE score of TCGA glioma samples were obtained by “ESTIMATE” package (version 1.0.13). This study compared the three scores between the two groups by rank sum test. Then the proportion of 22 immune cells was calculated using the Cell type Identification By Estimating Relative Subsets Of RNA Transcripts (CIBERSORT) algorithm (version 1.03) in the TCGA-glioma dataset. The proportion of each immune cell in each sample were gotten by CIBERSORT algorithm. The samples with *p* > 0.05 were excluded, and the proportions of immune cells in the remaining samples were displayed in box plots and bar charts. In addition, the immune cells between high and low risk groups were compared by the rank sum test and visualized by plotting box line plots using the “ggplot2” and “ggpubr” packages. Finally, the expression of 24 HLA related genes and nine immune checkpoints (LAG3, ICOS, TIGIT, CD274, PDCD1, IDO1, CD27, PDCD1LG2, and HAVCR2) were compared by rank sum test respectively.

### Single-cell functional analysis of model genes

In this study, 14 functions (angiogenesis, cell cycle, apoptosis, DNA damage, cell differentiation, DNA repair, EMT, cellular hypoxia, cancer cell invasion, metastasis, inflammation onset, proliferation, cell resting, and stem cell properties) of model genes were predicted using CancerSEA. Then the functional status of the model genes in glioma were queried and the significantly relevant functions were displayed.

## Data Availability

The original contributions presented in the study are included in the article/**Supplementary Material**, further inquiries can be directed to the corresponding author/s.

## Supplementary material

Data sheet 1 Highly expressed DEGs of glioma in TCGA database

Data sheet 2 Low expressed DEGs of glioma in TCGA database

Data sheet 3 Correlation of risk Score with KEGG pathway
